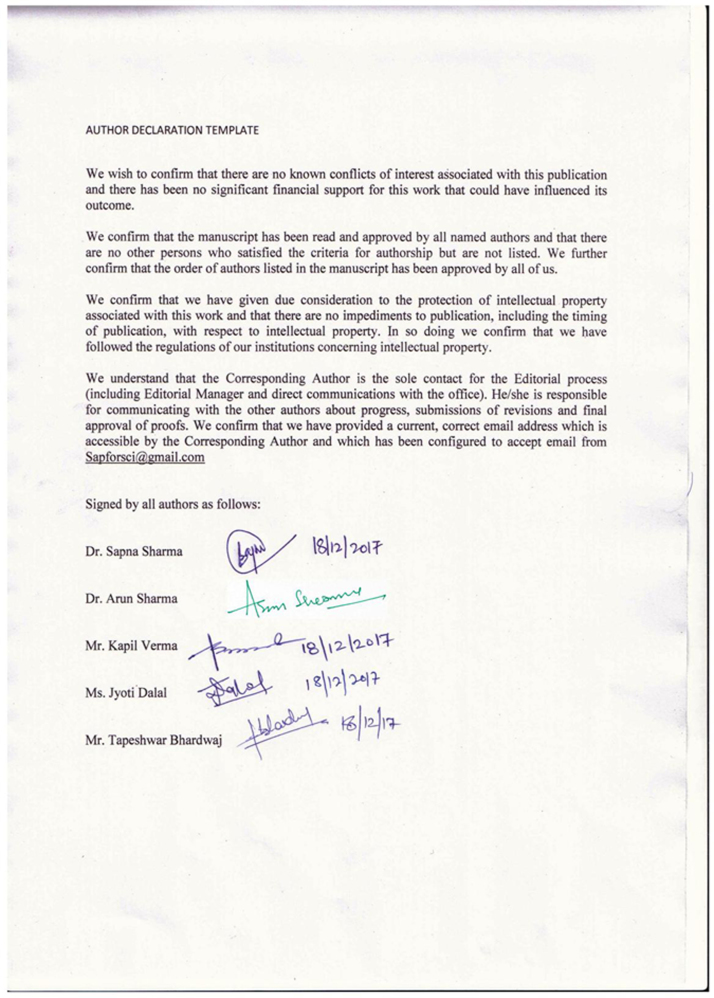# Data on haplotype diversity in the hypervariable region I, II and III of mtDNA amongst the Brahmin population of Haryana

**DOI:** 10.1016/j.dib.2018.01.011

**Published:** 2018-01-31

**Authors:** Kapil Verma, Sapna Sharma, Arun Sharma, Jyoti Dalal, Tapeshwar Bhardwaj

**Affiliations:** a Department of Genetics, Maharshi Dayanand University, Rohtak, Haryana 124001, India; b Govt. of Himachal Pradesh, Junga, Himachal Pradesh 173216, India

**Keywords:** Mitochondrial DNA, D-loop, Hypervariable regions, Forensic genetics

## Abstract

Human mitochondrial DNA (mtDNA) is routinely analysed for pathogenic mutations, evolutionary studies, estimation of time of divergence within or between species, phylogenetic studies and identification of degraded remains. The data on various regions of human mtDNA has added enormously to the knowledge pool of population genetics as well as forensic genetics. The displacement-loop (D-loop) in the control region of mtDNA is rated as the most rapidly evolving part, due to the presence of variations in this region. The control region consists of three hypervariable regions. These hypervariable regions (HVI, HVII and HVIII) tend to mutate 5–10 times faster than nuclear DNA. The high mutation rate of these hypervariable regions is used in population genetic studies and human identity testing. In the present data, potentially informative hypervariable regions of mitochondrial DNA (mtDNA) i.e. HVI (np 16024–16365), HVII (np 73–340) and HVIII (np 438–576) were estimated to understand the genetic diversity amongst Brahmin population of Haryana. Blood samples had been collected from maternally unrelated individuals from the different districts of Haryana. An array of parameters comprising of polymorphic sites, transitions, transversions, deletions, gene diversity, nucleotide diversity, pairwise differences, Tajima's D test, Fu's Fs test, mismatch observed variance and expected heterozygosity were estimated. The observed polymorphisms with their respective haplogroups in comparison to rCRS were assigned.

**Specifications Table**

**Table:** 

Subject area	Forensic Science
More specific subject area	Forensic Genetics
Type of data	Tables and Figures
How data was acquired	DNA from blood samples were extracted by using PCI method [1]. PCR amplification was carried out using SureCycler 8800 (Agilent Technologies, USA). All the amplified samples were cleaned before sequencing by using Post PCR Clean-up Kits (Thermo Scientific, USA) and sequencing were carried out using Applied Biosystems DNA Sequencer (Life Technologies, CA, USA). Analysis part were carried out by using Arlequin software version 3.5 (Computational and Molecular Population Genetics Lab, Zoological Institute, Switzerland) [2], Mega 7 [3] and HaploGrep 2 software (Medical University of Innsbruck, Austria) [4]
Data format	Analyzed
Experimental factors	Genomic DNA was extracted and amplified from the blood samples
Experimental features	PCR amplification of HVI, HVII and HVIII was carried out using SureCycler 8800 (Agilent Technologies, USA) and Sequencing were carried out using Applied Biosystems DNA Sequencer (by Life Technologies, CA, USA)
Data source location	Haryana (A northern state of India)
	Latitude: 29.0588°N
	Longitude: 76.0856°E
Data accessibility	The data is available with this article

**Value of the data**

• The data report will provide baseline information to any future evolutionary and genetic studies based on control region of mtDNA of the Brahmin population of Haryana.
• The data produced may be helpful in finding new mutations or polymorphisms which will prove quite useful in personal identification.
• It is also of special relevance to the investigative agencies in particular and society in general.
• In cases of mass disasters, it is common for the government agencies to provide compensations to the deceased family members. So the present data can also aid in the identification process of such cases.
• The present data will enhance the DNA database of Brahmin population, which can be used for calculating probabilities of match based on mtDNA.

## 1 Data

Table 1 describes the primer pairs used for amplifications of extracted samples.

**Table 1:** Details of the primers used for amplification of the Hypervariable Regions.

**Mt DNA region**	**Nucleotide Position**	**Primers**	**Primer Sequence (5′-3′)**	Tm Value	**Size (bp))**
HVI	16024–16365	L 15900 (F)	TACACCAGTCTTGTAAACC	49.1 °C	828
		H 00159 (R)	AAATAATAGGATGAGGCAGGAATC	52.5 °C	
HVII & III	73–576	L 00015 (F)	CACCCTATTAACCACTCACG	52.7 °C	585
		H 00599 (R)	TTGAGGAGGTAAGCTACATAA	50.3 °C	

Table 2 describes the PCR reaction mixture used for amplification of HVI, HVII and HVIII region.

**Table 2:** PCR reaction setup.

**S.No.**	**Chemical**	**Quantity**
1	10 × PCR buffer	2.5 µl
2	2 mM each dNTPs	2.5 µl
3	10 mM forward primer	1.5 µl
4	10 mM reverse primer	1.5 µl
5	5 U/µl Taq DNA polymerase	0.5 µl
6	D/DH2O	15.5 µl
7	50 ng of template DNA	1 µl

Table 3 summarizes the PCR cycling conditions adopted during experiment.

**Table 3:** PCR cycling conditions.

**Cycle step**	**Temperature**	**Time duration**
Hot start	95 °C	7 min
Denaturation	95 °C	15 s
Annealing	62 °C	30 s
Elongation	72 °C	1 min
End cycle Elongation	72 °C	10 s
Hold	4 °C	∞

Table 4 summarizes the molecular diversity as seen in the HVI region.

**Table 4:** Molecular diversity as seen in the HVI region.

**Population**	**Brahmin**
Sample size	66
No. of polymorphic sites	69
No. of observed transitions	62
No. of observed transversions	10
No. of observed substitutions	72
No. of observed indels	1
Nucleotide composition (%) C	33.29
T	22.42
A	33.00
G	11.29
Mean number of pairwise differences	5.78 ± 2.8
Heterozygosity/sample	0.016 ± 0.05
No of Haplotypes	51
Gene Diversity	0.986 ± 0.006
Nucleotide Diversity	0.0167 ± 0.009
Ss**2** of haplotype frequencies (RMP)	0.0285
Alleles Frequency (Mean ± S.D)	1.212 ± 0.437
Sum of square deviation	0.0011
Harpending's raggedness index	0.0081
Mismatch distribution observed mean	5.78
Mismatch observed variance	7.1
Tajima's D test	− 2.02
Fu's FS test	− 25.31

Table 5 summarizes the molecular diversity as seen in the HVII and HVIII region.

**Table 5:** Molecular diversity as seen in the HVII & HVIII region.

**Population**	**Brahmin**
Sample size	66
No. of polymorphic sites	58
No. of observed transitions	43
No. of observed transversions	13
No. of observed substitutions	56
No. of observed indels	5
Nucleotide composition (%) C	34.54
T	22.79
A	30.46
G	12.22
Mean number of pairwise differences	5.3 ± 2.59
Heterozygosity/sample	0.091 ± 0.12
No of Haplotypes	48
Gene Diversity	0.986 ± 0.005
Nucleotide Diversity	0.0104 ± 0.005
Ss**2** of haplotype frequencies (RMP)	0.285
Alleles Frequency (Mean ± S.D)	1.12 ± 0.34
Sum of square deviation	0.001
Harpending's raggedness index	0.0087
Mismatch distribution observed mean	5.3
Mismatch observed variance	6.31
Tajima's D test	− 2.03
Fu's FS test	− 25.45

Table 6 summarizes the molecular diversity as seen in the HVI + HVII + HVIII region.

**Table 6:** Molecular diversity as seen in the HVI + HVII + HVIII region.

**Population**	**Brahmin**
Sample size	66
No. of polymorphic sites	127
No. of observed transitions	105
No. of observed transversions	25
No. of observed substitutions	130
No. of observed indels	4
Nucleotide composition (%) C	34.04
T	22.64
A	31.48
G	11.84
Mean number of pairwise differences	11.09 ± 5.1
Heterozygosity	0.087 ± 0.1
No of Haplotypes	64
Gene Diversity	0.999 ± 0.002
Nucleotide Diversity	0.0129 ± 0.006
Ss**2** of haplotype frequencies (RMP)	0.016
Alleles Frequency (Mean ± S.D)	1.16 ± 0.26
Sum of square deviation	0.00034
Harpending's raggedness index	0.003
Mismatch distribution observed mean	11.09
Mismatch observed variance	13.73
Tajima's D test	− 2.09
Fu's FS test	− 24.47

Table 7 summarizes the frequency distribution of mtDNA haplotypes in Brahmin population.

**Table 7:** Frequency distribution of mtDNA haplotypes in Brahmins population.

**Number of times a haplotype repeated**	**Numbers of Haplotypes**		
	**HVI**	**HVII + HVIII**	**HVI + HVII + HVIII**
1	45	37	64
2	1	7	2
3	2	2	–
4	2	1	–
5	1	1	–
Total	51	48	64
**Random match probability**	0.028	0.028	0.016

Table 8 summarizes the sequence polymorphism and their respective haplogroups in the Brahmin population.

**Table 8:** Sequence polymorphism and their respective haplogroups in the Brahmin population.

Sample ID	HVI region	HVII region	HVIII region	Haplogroups
BR1	16154C 16206C 16230G 16311C	73G 263G 309.1C 315.1C	524.1AC	U2a1a
BR2	16129A 16223T 16343G	73G 189G 194T 195C 199C 204C 207A 263G 315.1C		W3a1+199
BR3	16189C 16223T	73G 212K 236C 263G 309.1C 315.1C	489C	M
BR4	16126C 16223T 16311C	73G 204C 263G 315.1C	482C 489C	M3a1+204
BR5	16167T 16172C 16318T	73G 151T 152C 263G 270G 275K 276M 315.1C	523d 524d	U7a1a
BR6	16129A 16223T 16362C	73G 263G 315.1C	489C	D4a
BR7	16169.1C 16189C 16193.1C 16223T 16274A	73G 152C 182T 195C 263G 309.1C 315.1C	447G 489C 523d 524d	M2b
BR8	16095T 16223T 16249C 16359C	73G 114T 146C 263G 309.1C 315.1C	489C	M34a1
BR9	16126C 16163G 16186T 16189C 16294T	73G 152C 195C 263G 309.1C 315.1C		T1a1'3
BR10	16046.1A 16183C 16189C 16193.1C 16223T 16362C	73G 195C 263G 299d 309.1CC 315.1C 373G		N9b
BR11	16069T 16126C 16145A 16172C 16222T 16261T	73G 242T 263G 295T 315.1C	462T 489C	J1b1a1
BR12	16309G 16318T	73G 151T 152C 263G 315.1C	523d 524d	U7a
BR13	16145A 16176T 16209C 16223T 16261T 16311C	73G 152C 263G 315.1C	489C	M4A
BR14	16126C 16294T	73G 194T 200G 263G 309.1C 315.1C		T2d1b
BR15	16260T 16261T 16286T 16311C 16319A 16362C	73G 146C 152C 263G 315.1C		R7b1a
BR16	16111T 16172C 16184T 16189C 16223T 16274A 16295T	73G 263G 309.1C 315.1C	489C	M37e
BR17	16189C 16223T	73G 195C 263G 315.1C		N9b
BR18	16104T 16184T 16223T 16311C	73G 150T 195C 198T 199C 207A 263G 309.1C 315.1C	489C 523d 524d	M66b
BR19	16172Y 16220C 16264T 16292T	263G 309.1CC 315.1C	456T	HV12a
BR20	16209C 16239T 16352C 16353T	73G 146C 152C 234G 263G 309.1C 315.1C		U2b2
BR21	16223T 16311C	73G 114T 263G 309.1C 315.1C 357C 361.1A	489C 524.1AC	M4"67+16311
BR22	16309R 16318C	73G 151T 152C 263G 309.1C 315.1C	489C 523d 524d	M37+152+151
BR23	16309G 16318T	73G 151T 152C 263G 309.1C 315.1C	523d 524d	U7a
BR24	16178C 16223T 16288C 16293T	73G 152C 204C 207A 263G 315.1C	489C 513A	M33d
BR25	16243C	73G 263G 309.1C 315.1C	489C	M49d
BR26	16129A 16223T 16291T	73G 152C 263G 315.1C 334C	489C	M5b2
BR27	16188T 16223T 16231C 16362C	73G 146C 152C 263G 309.1C 315.1C	461T 489C	M6a1b
BR28	16175G 16223T 16234T	73G 195A 263G 315.1C	489C 523d 524d	M30+16234
BR29	16309G 16318T	73G 151T 152C 222A 263G 309.1C 315.1C	476A 523d 524d	U7a
BR30	16126C 16223T 16311C	73G 98A 146C 152C 207S 263G 315.1CC 338T	523d 524d	L3h1
BR31		73G 117A 146C 263G 309.1C 315.1C	482C 489C	M3
BR32	16309G 16318T	73G 152C 153G 195C 263G 315.1C	523d 524d	U7
BR33	16126C 16163G 16186T 16189C 16294T	73G 152C 263G 309.1C 315.1C		T1a+152
BR34	16126C 16223T 16311C	73G 204C 263G 315.1C	482C 489C	M3a1+204
BR35	16111T 16184T 16189C 16223T 16274A 16295T	263G 315.1C		M37e
BR36	16126C 16181G 16209C 16304C 16362C	73G 152C 263G 309.1C 315.1C		R9b2
BR37	16051G 16206C 16311C	73G 191.1A 194T 263G 315.1C		U2a
BR38	16169.1C 16183C 16189C 16193.1C 16223T 16258C 16274A 16319A	73G 146C 152C 182T 195C 263G 315.1C 325T	447G 489C 523d 524d	M2b
BR39	16224C 16311C	73G 132T 146C 152C 263G 309.1CC 315.1C 324T		K2a5
BR40	16223T 16234T	73G 195A 263G 315.1C	489C 523d 524d	M30+16234
BR41	16129A	263G 309.1C 315.1C		H1e+16129
BR42	16111T 16169.1C 16189C 16223T 16274A 16319A 16320T	73G 152C 182T 263G 315.1C	447G 471C 523d 524d 530T	M2b1b
BR43	16111T 16184T 16223T 16266T 16296T	73G 78T 120G 152G 167G 180G 263G 315.1C	489C 523d 524d	M7b2
BR44	16223T 16256T 16311C 16362C	73G 263G 309.1C 315.1C	489C	M43a1
BR45	16183C 16189C 16193.1C 16223T	73G 153G 195C 225A 226C 263G 315.1C		X2b+226
BR46	16179d 16223T	73G 146C 242T 263G 295T 315.1C	462T 489C 523d 524d	M7c
BR47	16223T 16327A	73G 125G 146C 152C 195C 263G 315.1C	489C 523d 524d	M24
BR50	16126C 16163G 16186T 16189C 16294T	73G 146C 152C 263G 315.1C	523d 524d	T1a+152
BR51	16189C 16223T	73G 194T 263G 315.1C		N9b
BR52	16145A 16176T 16223T 16261T 16311C	73G 263G 309.1C 315.1C	482C 489C	M4a
BR53	16126C 16170G 16223T 16311C	73G 146C 152C 263G 309.1C 315.1C	461T 489C 573.1CC	M6
BR54	16051G 16254G	73G 85C 180C 216G 263G 309.1C 315.1C		U2
BR55	16069T 16126C 16145A 16222T 16261T 16288C 16362C	73G 195C 263G 309.1C 315.1C	462T 489C	J1b
BR56	16092C 16126C 16223T 16311C	73G 263G 315.1C	482C	M3
BR57	16188G 16223T 16270T 16274A 16290T 16291T 16319A 16352C	263G 315.1C		H1ba
BR58		73G 146C 263G 315.1C	489C	M7c
BR59	16126C 16248T 16292T 16294T 16296T 16325C 16327T	73G 204C 263G 315.1C	447G 489C	T2c1
BR60		73G 263G 309.1C 315.1C	489C	M
BR61	16223T 16234T	73G 263G 309.1C 315.1C		N
BR62	16051G 16129C 16182C 16183C 16189C 16362C	73G 195A 263G 309.1C 315.1C	489C 523d 524d	U2e
BR63		152C 215G 263G 309.1C 315.1C		H1+152
BR64	16223T	152C 215G 263G 309.1C 315.1C		N1a
BR65	16126C 16223T 16311C	73G 152C 217C 263G 309d 315.1C 394T	508G	L3h1
BR66	16069T 16126C 16145A 16172C 16222T 16261T 16319A	73G 146C 242T 263G 295T 315.1C	462T 489C 523d 524d	J1b1a1+146
BR67	16126C 16223T 16311C	73G 195A 263G 309.1CC 315.1C	489C 523d 524d	M30
BR69	16126C 16223T 16311C 16320T	73G 152C 263G 309.1C 315.1C		L3h1

Table 9 GenBank accession numbers for Brahmin Population (Supplementary Table).

## 2 Experimental design, materials and methods

### 2.1 Sample collection and genomic DNA extraction

The present study was completed in different phases. The first phase comprised of blood samples collection followed by the second phase, which involved the molecular biology procedures for DNA extraction, PCR amplification, PCR clean-up and sequencing. The last phase consisted of statistical analysis and interpretation of the data generated.

Blood samples had been collected from maternally unrelated individuals from nearly all districts of Haryana belonging to the ethnic group of Brahmins, after following proper ethical guidelines. Total genomic DNA was extracted from the samples using the Phenol-Chloroform method [1]. The extracted DNA was checked for its quality on 0.8% agarose gel and quantity was checked on the Nanodrop (Thermo scientific, USA).

### 2.2 PCR amplification

The three hypervariable regions, i.e. the HVI lying between np16024 and 16365, HVII lying between np 73–340 and HVIII lying between np 438–576 were amplified using both forward and reverse primers. The primer pairs used by the Brandstatter et al. [5] were used for amplifications. They were synthesized at IDT (Integrated DNA Technologies (IDT), USA) (Table 1). Two sets of PCR reactions were used for each sample, i.e. one for HVI region alone and the other amplified both the HVII and HVIII regions together. All controls, i.e., control and − ve extraction controls and amplification controls along with a reagent blank control were used during the experiments. Controls were used to ensure that no contamination was present at any stage during all the experiments. The PCR reaction was carried out in a final volume of 25 µl given in Table 2. PCR was performed on (SureCycler 8800, Agilent Technologies, USA). The PCR cycling conditions used are given in Table 3. After PCR amplification, the amplified product was visualized on 1.6% agarose (Sisco Research Laboratory, India) gel. GeneRuler 100 bp ladder (Thermo scientific, USA) was used for reading the size of the amplified product. After electrophoresis, the gel was visualized under Gel Documentation System (Alpha Innotech).

### 2.3 Post PCR cleanup

All the samples were cleaned before sequencing by using Post PCR Clean-up Kits (Thermo Scientific, USA) to remove the PCR inhibitors, primer-dimer formation and impurities present in the template.

### 2.4 Sequencing

The sequencing was carried out in the Xcelaris Genomic Labs by using the ABI BigDye Terminator Cycle Sequencing Kit on ABI 3700 Genetic analyzer (Applied Biosystems). All the samples were sequenced with the same primers used in PCR amplification for HVI, HVII & HVIII regions. An additional primer (16410R- GAGGATGGTGGTGGTCAA) has also been used for hyper variable region I in samples where slippage due to ‘C’ stretch was observed.

### 2.5 Statistical analysis

**Fig. 1:**
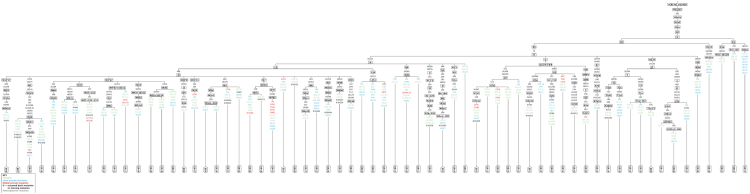
Phylogenetic tree of haplogroups including all related polymorphisms relative to the rCRS for Brahmin Population.

The interpretation of the HVI, HVII and HVIII chromatogram was done as per the guidelines to improve the quality of the data [6], [7], [8], [9]. The sequences were matched and aligned with the revised Cambridge reference sequences (rCRS) [10] by using Mega 7 [3]. The coding for heteroplasmic sites was done according to the IUPAC codes in the interpretation guideline to interpret the mtDNA data analysis [8]. Diversity indices and differentiation tests were computed. The gene diversity was calculated according to Tajima [11]. Population pairwise differences were calculated by using genetic distances [12]. Nucleotide diversity, haplotype diversity, mean pairwise difference, number of haplotypes, mismatch distributions, Harpending's raggedness index, Tajima's D test and Fu's Fs statistics were calculated by using Arlequin software version 3.5.1.2 [2] as shown in Table 4, Table 5. A random match probability (RMP) was calculated according to Stoneking et al. [13] (Table 6). Haplogroups classification and phylogenetic tree was performed by using HaploGrep 2 software [4] as shown in Table 8 and Fig. 1.

## Figures and Tables

**Fig. S1:**