# Supplementary material for: Data on haplotype diversity in the hypervariable region I, II and III of mtDNA amongst the Brahmin population of Haryana
Source: Data Brief. 2018 Jan 31;17:305–13. doi: 10.1016/j.dib.2018.01.011 (PMC5988218; doi:10.1016/j.dib.2018.01.011)
Supplement: Supplementary file 1 — Supplementary material [file mmc2.docx]

**Supplementary Table:**

**Table 9**: GenBank sequences Accession numbers for Brahmin population

| MG431877 | MG431899 | MG431921 |
| --- | --- | --- |
| MG431878 | MG431900 | MG431922 |
| MG431879 | MG431901 | MG431923 |
| MG431880 | MG431902 | MG431924 |
| MG431881 | MG431903 | MG431925 |
| MG431882 | MG431904 | MG431926 |
| MG431883 | MG431905 | MG431927 |
| MG431884 | MG431906 | MG431928 |
| MG431885 | MG431907 | MG431929 |
| MG431886 | MG431908 | MG431930 |
| MG431887 | MG431909 | MG431931 |
| MG431888 | MG431910 | MG431932 |
| MG431889 | MG431911 | MG431933 |
| MG431890 | MG431912 | MG431934 |
| MG431891 | MG431913 | MG431935 |
| MG431892 | MG431914 | MG431936 |
| MG431893 | MG431915 | MG431937 |
| MG431894 | MG431916 | MG431938 |
| MG431895 | MG431917 | MG431939 |
| MG431896 | MG431918 | MG431940 |
| MG431897 | MG431919 | MG431941 |
| MG431898 | MG431920 | MG431942 |
